# The Influence of Nanofiller Shape and Nature on the Functional Properties of Waterborne Poly(urethane-urea) Nanocomposite Films

**DOI:** 10.3390/polym12092001

**Published:** 2020-09-02

**Authors:** Milena Špírková, Jiří Hodan, Rafał Konefał, Luďka Machová, Pavel Němeček, Aleksandra Paruzel

**Affiliations:** Institute of Macromolecular Chemistry CAS, Heyrovského nám. 2, 162 06 Prague, Czech Republic; hodan@imc.cas.cz (J.H.); konefal@imc.cas.cz (R.K.); machova@imc.cas.cz (L.M.); nemecek@imc.cas.cz (P.N.); gawelczyk@imc.cas.cz (A.P.)

**Keywords:** waterborne poly(urethane-urea) dispersion, nanocomposite, film, degradability, starch, hydroxyapatite

## Abstract

A series of waterborne polycarbonate-based poly(urethane-urea) nanocomposite films were prepared and characterized. An isocyanate excess of 30 mol% with respect to the hydroxyl groups was used in the procedure, omitting the chain-extension step of the acetone process in the dispersion preparation. The individual steps of the synthesis of the poly(urethane-urea) matrix were followed by nuclear magnetic resonance (NMR) spectroscopy. The nanofillers (1 wt% in the final nanocomposite) differed in nature and shape. Starch, graphene oxide and nanocellulose were used as representatives of organic nanofillers, while halloysite, montmorillonite, nanosilica and hydroxyapatite were used as representatives of inorganic nanofillers. Moreover, the fillers differed in their shape and average particle size. The films were characterized by a set of methods to obtain the tensile, thermal and surface properties of the nanocomposites as well as the internal arrangement of the nanoparticles in the nanocomposite film. The degradation process was evaluated at 37 °C in a H_2_O_2_ + CoCl_2_ solution.

## 1. Introduction

Polyurethane (PU) materials, PU blends and composites are important materials and are extensively studied and used as polymer systems [1,2,3]. The popularity of polyurethane nanocomposites is caused mainly by the simplicity of tuning their functional properties. The choice of nanofillers, for example, clay [4], SiO_2_ [5], graphene oxide [6], cellulose [7], starch [8], nanosilver [9], nanogold [10], TiO2 [11] or Fe_3_O_4_ [12], is miscellaneous. PU can be used in a wide range of applications in different industries, especially in the construction, automotive and shoe industries [1,2,3,13,14]. Polyurethane-based materials are also used in medical and biomedical applications, either as long-term implanted devices, vascular prostheses, catheters, prosthetic valve leaflets or as biodegradable materials, used in controlled drug delivery systems, such as cardiovascular grafts, temporary scaffolds in tissue engineering and so forth. [15,16,17,18,19,20,21,22,23].

The development of current PU and related systems is mostly focused on special low-quantity PU products with tailored functional properties. PU used as thin films and coatings is related to the preparation and application of waterborne PU dispersions [4,5,6,7]. The importance of PU dispersions is reinforced by the strong regulation of reducing volatile organic compounds (VOCs) and hazardous air pollutants (HAPs). PU dispersions are nontoxic and do not pollute the air.

Waterborne polyurethane dispersions (WPUDs) as spherical submicron-sized particles are used as precursors for the preparation of 2D-systems—coatings and adhesives [24,25,26]. WPUDs can be used alone or mixed with water-dispersed admixtures and the whole process is finalized by water (solvent) evaporation. Their use in the textile industry was the earliest and most extended application of WPUDs but they can also be employed as coatings and adhesives for paper, leather, metal, plastic and wood [24,25,26,27,28,29]. The current use of WPUDs is shifting towards medical applications, for example, as biodegradable materials [30,31]. As the films/coatings of WPUDs are thin, their application often requires the synthesis of specific (sometimes relatively expensive) biomaterials with targeted properties.

Waterborne PU dispersions are mostly prepared using the ‘acetone method’ [27,32,33], which consists of four steps. The preparation involves prepolymerization, neutralization, chain extension and phase inversion processes. Recently, we prepared stable polycarbonate-based WPUDs by an innovative ‘acetone method’ without a chain-extension step [34]. This technique enables the preparation of films with variable functional properties using different excesses of isocyanates with respect to the total number hydroxyl groups. The excess isocyanate reacts with the water during the last phase-inversion step into urea, eventually resulting in biuret structures. WPUDs are formed from branched, eventually crosslinked, poly(urethane-urea) chains. This technique can lead to materials with substantially improved tensile properties compared with films prepared from WPUDs chain-extended by 1,4-butandiol with linear PU chains [35]. However, the urethane:urea/biuret ratio must be balanced to obtain mechanically strong films. Under a given preparation procedure and with given starting components, the mechanical properties depend, for example, on the macrodiol regularity. Films with ‘optimal’ tensile properties are prepared with approximately 30 mol% isocyanate excess made from regular macrodiol T4672 [34] and with approximately 40 mol% for systems with irregular T5652 macrodiol [36].

This aim of this study is to prepare different waterborne PU nanocomposites containing 1 wt% of nanofiller. The polyurethane matrix is based on regular macrodiol G4672 and 30 mol% isocyanate excess. The macrodiol G4672 was used in our study for the first time. Organic (nanocellulose, starch, graphene oxide) and inorganic (montmorillonite, SiO_2_, halloysite and hydroxyapatite) nanofillers differ in particle size and shape.

To the best of our knowledge, the set of starting materials used for the poly(urethane-urea) dispersion preparation by the novel technique (reported in our recent publication [34]), followed by nanocomposite preparation and multiscale characterization (accelerated in vivo degradation tests included), have not yet been described. Two purposes for the study were—(i) the individual steps of the poly(urethane-urea) particle synthesis were checked by proton nuclear magnetic resonance (^1^H NMR) spectroscopy and (ii) the nanocomposite film synthesis and characterization was concentrated on the selection of the most prospective combination polyurethane-urea (PUU) matrix-nanofiller for top coating applications potentially usable in the human-body conditions.

## 2. Experimental

### 2.1. Materials

*Polyurethane preparation*—Aliphatic polycarbonate macrodiol (PCD, trademark G4672) was kindly provided by Asahi Kasei Chemical Corporation, Tokyo, Japan. G4672 is a telechelic linear oligomer end-capped with hydroxyl groups consisting of butylene (C4) and hexylene (C6) units connected by carbonate groups (C6/C4 molar ratio = 7:3). The detailed NMR analysis of G4572 is shown in the Supplementary results (in the NMR spectroscopy section and in Figure S1). 1,6-Diisocyanatohexane (HDI), 1,4-butanediol (BD), 2,2-bis(hydroxymethyl) propionic acid (DMPA) and triethylamine (TEA) were obtained from Sigma Aldrich, Steinheim, Germany. Dried acetone (max. 0.0075% H_2_O) was supplied by Merck KGaA (Darmstadt, Germany). Dibutyltin dilaurate (DBTDL, Sigma Aldrich, Steinheim, Germany) was used as a catalyst in the form of a 10 wt% Marcol oil solution (mixture of liquid saturated hydrocarbons).

*Nanofillers*—Nanofillers were commercially available products with the exception of graphene oxide, which was prepared in IMC by the Hummers method [37]. All of the nanofillers were obtained either in the form of a water suspension, as for nanocellulose (CNC), hydroxyapatite (HAp), nanosilica (SiO_2_), graphene oxide (GO) or in the form of powder, as for Cloisite^®^ Na^+^ (MNa), starch (St) and halloysite (HALL). The producers and basic nanofiller characterization are shown in Table S1.

### 2.2. Preparation Procedure

#### 2.2.1. Polyurethane-Urea Water Dispersion (PUUD)

Waterborne poly(urethane-urea) dispersion (WPUUD) was prepared via an acetone process without a chain-extension step [34] keeping a PCD-to-DMPA molar ratio of 1:1 and the [NCO]/[OH]_total_ ratio equal to 1.3 (where [OH]_total_ = [OH]_PCD_ + [OH]_DMPA_), which corresponds to a 30 mol% excess of isocyanate groups with respect to the total content of reactive hydroxyl groups.

**Figure 1:**
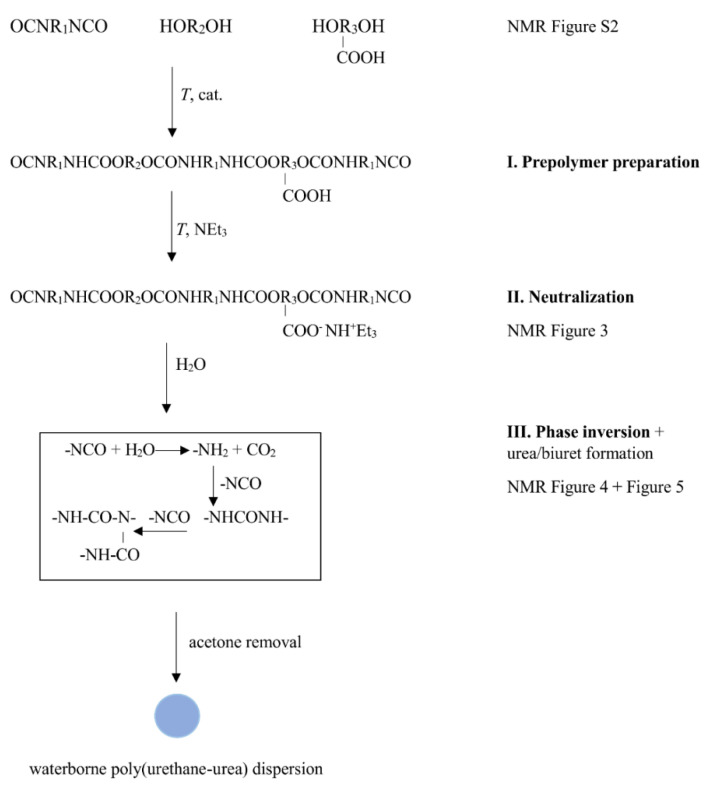
Scheme of waterborne poly(urethane-urea) dispersion preparation.

The simplified scheme of the preparation procedure is shown in Figure 1. The process consists of prepolymer preparation, neutralization of carboxylic groups and a phase inversion connected with the reaction of excess isocyanate groups with water.

Given amounts of G4672, DMPA, HDI, catalyst (0.05 mol% of DBTDL per mol of NCO groups) and acetone were mixed at 700 rpm at 60 °C for 5 h (I. Prepolymerization step). Triethylamine neutralized the carboxylic groups in DMPA under continuous stirring at 55 °C for 30 min (II. Neutralization step). Finally, water was gradually added to the polyurethane solution in acetone (III. Phase inversion and excess isocyanate + water reactions). Organic solvent-free PUUDs were obtained after removal of acetone under reduced pressure. The solid content in the final waterborne PUUD was 30 ± 1 wt%.

#### 2.2.2. Nanocomposite Preparation

All the nanocomposites contained 1 wt% of the nanofiller in the film. The nanofillers were added to the WPUUD (placed in closed vials equipped with a magnetic stirrer) either in the form of a powder (halloysite, montmorillonite, starch) or in the form of a water dispersion/suspension (hydroxyapatite, nanosilica, graphene oxide, nanocellulose). The methods for nanofiller mixing in the WPUUD differed but the combination of mixing in an ultrasonic bath and stirring with a magnetic stir bar was used in all cases. Moreover, shaking was also used in the case of graphene oxide and nanocellulose. The time of the individual processes was adjusted to achieve a high nanofiller dispersion in the WPUUD without any noticeable clusters. The total time of dispersion/nanofiller mixing is shown in Table S1 (7th column). Ambient temperature was used in all cases with the exception of starch, for which the starch/WPUUD mixture was heated for 20 min at 65 °C to achieve starch solubility in the WPUUD.

**Figure 2:**
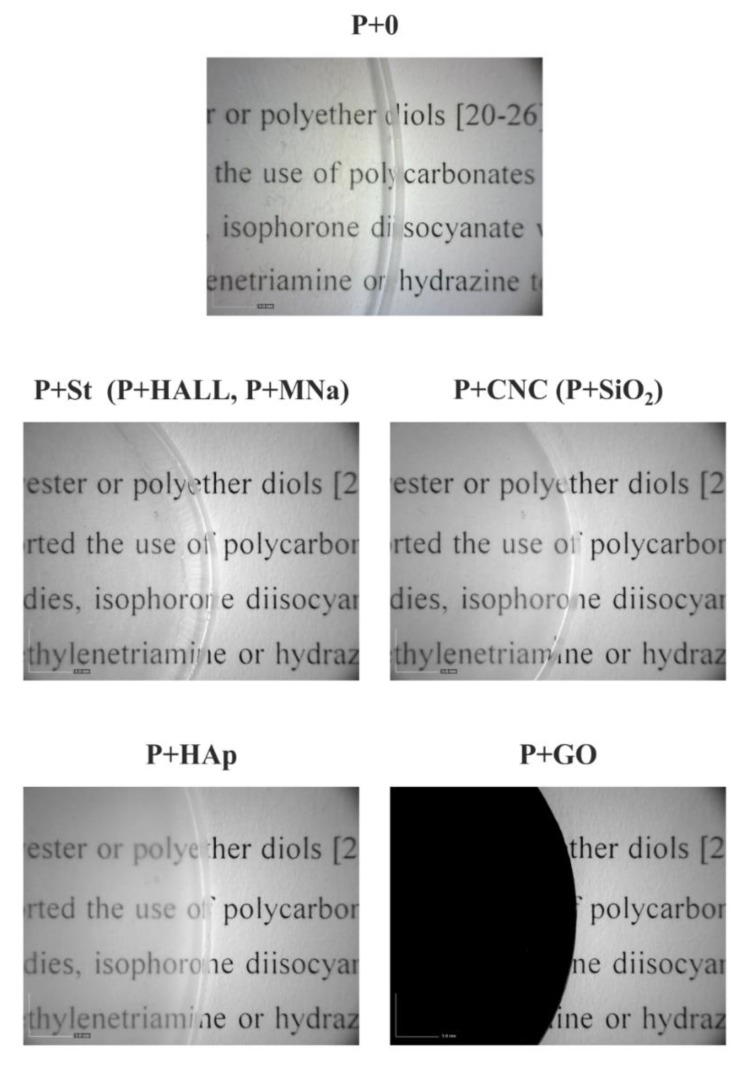
Optical microscopy of poly(urethane-urea)-based films; magnification 10 ×. (Films in parentheses are almost identical to the selected films).

The WPUUD/nanofiller mixtures were subsequently added to Teflon molds. Water was slowly evaporated, first at ambient temperature for 3 days and then at 50 °C for 20 h and finally, the mixtures were dried in vacuum for 1 h at 50 °C. The final dry film thickness was 300 ± 30 µm in all cases. The resulting films were mostly transparent or only slightly opaque (nanocomposite with hydroxyapatite). The only exception was the black nanocomposite containing graphene oxide. Figure 2 shows images of selected films acquired with an optical microscope at a magnification of 10×. The films not shown (in parentheses) had almost identical transparencies to the selected films.

### 2.3. Methods of Characterization

*NMR spectroscopy.*^1^H NMR spectra were recorded with a Bruker Avance III 600 spectrometer operating at 600.2 MHz in 5 mm NMR tubes using deuterated dimethylformamide (DMF) as the external solvent (in a capillary) at 25 °C. The width of the 90° pulse was 10 μs, the relaxation delay was 10 s and the acquisition time was 2.18 s and 16 scans were performed.

*Static mechanical properties* were measured according to test method ISO 527 at room temperature with a cross-head speed of 10 mm.min^−1^ with a 100 N load cell on an Instron model 6025/5800R (Instron Limited, High Wycombe, UK). Dumbbell-shaped specimens of 0.3 mm thickness (the total specimen length was 35 mm; the length and width of the narrowed part were 12 and 2 mm, respectively) corresponded to the ISO 527-2/5B type.

*Transmission electron microscopy* (TEM) was used to visualize the dispersion of the filler in the nanocomposites and the spherical semicrystalline formations (spherulites) in amorphous poly(urethane-urea). TEM micrographs were obtained using a transmission electron microscope (Tecnai G2 Spirit Twin 12; FEI Company, Brno, Czech Republic) in bright-field mode at an accelerating voltage of 120 kV. The ultrathin sections (thickness ~ 60 nm) were prepared using an ultramicrotome equipped with a cryo-attachment (Ultracut UCT, Leica, Vienna, Austria). The temperatures of the knife and samples were −40 °C and −80 °C, respectively.

*Thermogravimetric analysis* (TGA) was performed with a Pyris 1 TGA thermogravimetric analyzer (PerkinElmer, Waltham, Massachusetts, USA). The samples were heated at a rate of 10 °C·min^−1^ from 30 to 600 °C under a nitrogen atmosphere.

*Fourier-transform infrared spectroscopy* (FTIR) spectra of the PU samples were recorded using a Perkin Elmer Spectrum 100 equipped with a universal ATR accessory with a diamond crystal. Spectra were recorded in the wavenumber range of 650–3500 cm^−1^. 

*Degradation experiments* were performed by incubating the samples in 20% H_2_O_2_ + 0.1 M CoCl_2_ for up to 21 days at 37 °C. If necessary, the degradation medium was changed after one week. Then, the samples were washed, dried and measured under the same conditions as the untreated samples.

## 3. Results and Discussion

The aims of the current study were (i) to evaluate the individual processes occurring during the WPUUD preparation by innovative techniques and (ii) to screen the functional properties of waterborne nanocomposites with 1 wt% of the nanofiller differing in nature and shape. The results are further evaluated with respect to the potential practical use of the material as stable/degradable top coating used in the human-body conditions.

### 3.1. Dispersion Formation 

#### NMR Spectroscopy

High-resolution NMR spectroscopy was used to monitor and follow the processes during the formation of the poly(urethane-urea) dispersions.

**Figure 3:**
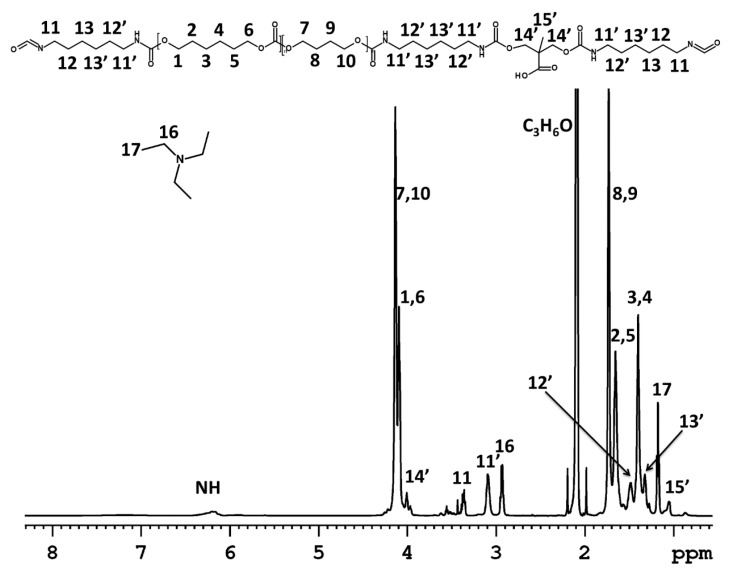
Proton nuclear magnetic resonance (^1^H NMR) spectrum of the reaction mixture measured at 22 °C after polyaddition and neutralization reactions using deuterated dimethylformamide (DMF) as the external solvent (in a capillary).

**Figure 4:**
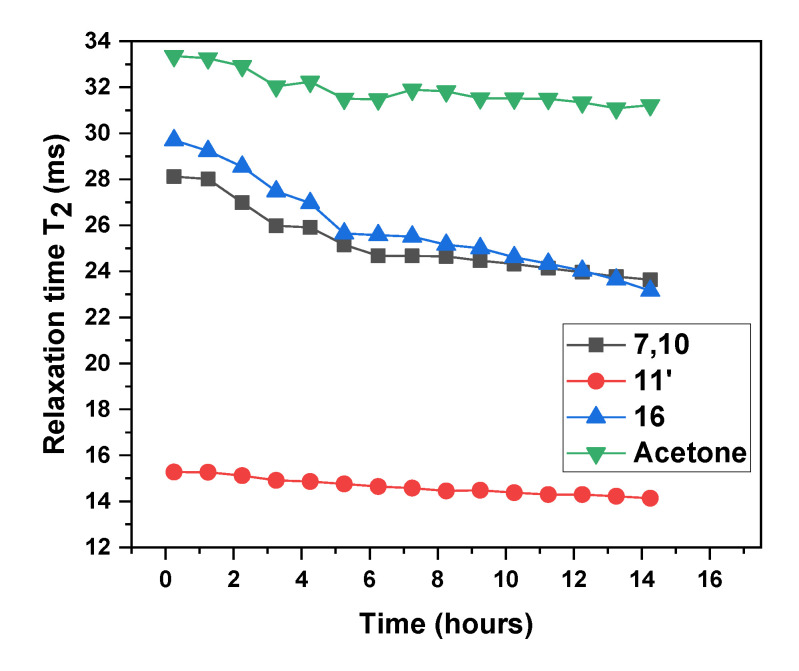
Time dependence of the *T*_2_ relaxation times of the polyurethane mixture in acetone measured after the polyaddition and neutralization reactions (calculated from the linewidths using the relation *T*_2_ = (*π*Δ*ν*)^−1^).

**Figure 5:**
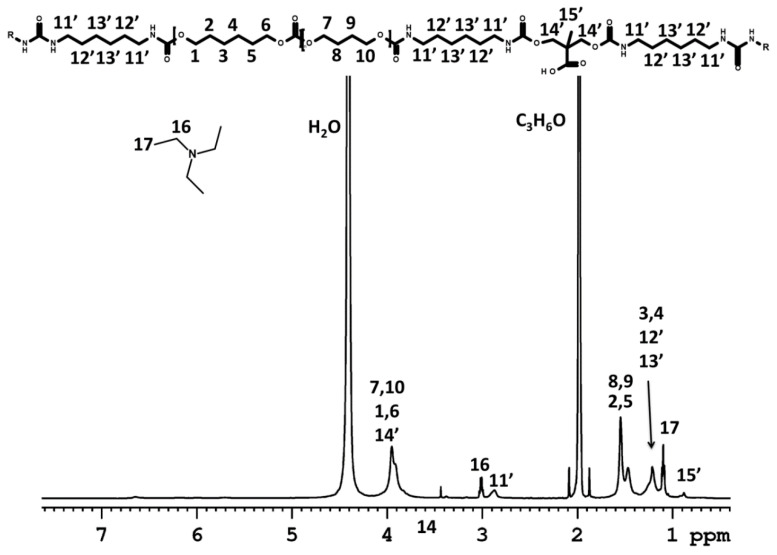
^1^H NMR spectrum of the product mixed with water measured at 22 °C using deuterated DMF as the external solvent (in a capillary).

^1^H NMR spectra were used for the structural characterization of the products during the WPUUD preparation. ^1^H NMR spectra of the reaction mixtures were recorded for (i) the original polycarbonate diol composed of hexane- and butane-repeating units (Figure S1); (ii) the mixture of PCD, HDI, DMPA and the catalyst in acetone before starting the polyaddition (Figure S2); (iii) the mixture of PCD, HDI, DMPA, the catalyst and TEA in acetone after 5 h of polyaddition and ½ h of neutralization (Figure 3); and (iv) the mixture of PCD, HDI, DMPA, the catalyst and TEA after 5 h of polyaddition; ½ h of neutralization; overnight storage (Figure 4); and 3 h after water addition (Figure 5).

The ^1^H NMR spectra proved the reaction scheme shown in Figure 1. Figure 3 shows the ^1^H NMR experiment after the reaction of the hydroxyl groups with the isocyanates and after neutralization of the carboxyl groups by TEA. The spectrum shows the new signals of the TEA methylene (**16**) and methyl (**17**) protons at δ ≈ 2.90 ppm and δ ≈ 1.15 ppm, respectively, compared to the NMR spectra shown in Figure S2. The ^1^H NMR spectrum confirmed the successful polyaddition reaction, as indicated by the appearance of a resonance peak related to the N**H** protons (at δ ≈ 6.30 ppm) and by the shift of the signals of HDI and DMPA (see Figure S1 signals marked with ‘). In the case of HDI, the signals related to the methylene protons (**11,’ 12,’ 13’**) were shifted from δ ≈ 3.38, 1.65 and 1.40 ppm to δ ≈ 3.10, 1.48 and 1.33 ppm, respectively. In addition, there was a significant shift in the signal associated with the methylene protons of DMPA (**14’**) from δ ≈ 3.70 ppm to δ ≈ 4.00 ppm. All of the abovementioned changes as well as the disappearance of PCD end-groups prove that the polyaddition reaction led to the formation of polyurethane.

The next experiment was performed to verify the processes occurring in the systems taking into account that the phase transition of the neutralized prepolymer did not proceed immediately but after some period of time (overnight in our case). To characterize possible changes during storage, the sample was stored in a magnet overnight and a series of ^1^H NMR spectra over a 1 h time period at 22 °C were collected and evaluated. Small changes, such as slight broadening of the signals or a small shift in the TEA methylene signal (**16**) *δ* with increasing time, were observed. Therefore, the *T*_2_ relaxation times of the methylene groups of polyurethane, TEA and acetone (**7, 10, 11,’ 16, C_3_H_6_O)**, which are close to: C=O, NH or N groups (these groups can create hydrogen bonds), were calculated from the linewidths using the relation *T*_2_ = (*π*Δ*ν*)^−1^. The results in Figure 4 show the time dependence of the *T*_2_ relaxation times. The low values of the *T*_2_ relaxation times (from ≈15 to 30 ms) calculated from the first experiment indicated that the polymer chains, acetone (neat solvent was also measured and calculated; *T*_2_ = 88 ms) and TEA were restricted in their mobility. This phenomenon is caused by strong intermolecular interactions, hydrogen bonds between NH and carbonyl groups and between the compounds in the reaction mixture (acetone and prepolymer chains). Moreover, the low mobility of TEA (similar to polyurethane prepolymer) suggests that its molecules effectively interacted with carboxylic functional groups originating from DMPA to form the strong complex –COO^−^NH^+^(C_2_H_5_)_3_. This formation was also proven by the small shift of the methylene group (**16**) over time from *δ* = 2.92 to 2.90 ppm observed in the literature by Kohler et al. [38]. The *T*_2_ values of all the tested units in the mixture decreased with increasing time, which confirmed the gradual mobility decrease; this phenomenon was more pronounced for protons belonging to macrodiol (**7**,**10**) and TEA (**16**) than for units belonging to isocyanate (**11’**). The proton mobility in the tested units decreased in the order isocyanate, macrodiol ≈ TEA and acetone. It is important to note the acetone behavior—acetone after neutralization was not considered a ‘free’ solvent (*T*_2_ of 33 ms in the reaction mixture; *T*_2_ of 88 ms of neat acetone) and its mobility slightly decreased with increasing exposure time. These experiments revealed that the system after neutralization is still ‘alive’ due to not only strong physical and ionic interactions between the polyurethane chains but also weak interactions between the polyurethane chains and acetone. All the physical interactions can cause gelation of the reaction mixture, thereby limiting the possibility of preparing waterborne dispersions. Therefore, water needs to be added to enable phase inversion after neutralization without delay.

In the last step of the procedure, water was added to the mixture and ^1^H NMR experiments were performed after the phase inversion was measured (see Figure 5). In the spectrum, the signals (broader than before water addition, see Figure 3) of the polymer were observed. This is related to the fact that in a mixture of a good solvent (acetone) and poor solvent (water), hydrophobic polymer chains are more restricted in mobility because they form nanoparticles. Moreover, water addition results in the generation of urea [39], which was confirmed by the disappearance of the signal of methylene protons OCN-**CH_2_**- **(11)** (see Figure 3).

The series of NMR experiments thus confirmed the anticipated macrodiol structure and the reaction scheme presented in Figure 1; in addition, the results led to an explanation why some preparation procedures can result in gelation of the reaction mixture before or during the phase transition.

### 3.2. Film Formation

The formation of continuous film from waterborne submicron PUU particles is the complex process proceeding in several steps—the PUU particles are in water isolated first but during slow water evaporation the process continues via the close contact of the particles, their deformation, coalescence and it finalizes in the compact film formation. While the chemical composition of PUU material is given by the preparation protocol, the functional properties of the final film depend significantly on the strong physical bonds and other dipole-dipole interactions being mainly formed during and after water evaporation. The final material is thermoplastic, composed of branched, eventually slightly crosslinked structures (see Figure 1).

In the case of nanocomposites prepared by mixing of waterborne PUU dispersion with the nanofiller, the film processing is even much more complicated. While the neat compact film is homogeneous in the composition, all the nanocomposites are heterogeneous, composed of the PUU matrix and the filler differently spread in the matrix. Through the filled films contain identical mass amount of the nanofiller (1 wt%), they noticeable differ in the volume fractions of the filler (in factor up to 3.5) but they significantly differ in the shape (aspect ratio of fillers spans from 1 for spheres to ca 350 for graphene oxide sheets). In this way, the characteristics will be further discussed with regards mainly to their aspect ratio (shape).

### 3.3. Tensile Properties

Our previous results obtained for polycarbonate-based polyurethanes revealed [34,40,41,42] that tensile characteristics are the most sensitive method reflecting changes in the sample composition and preparation procedure. Therefore, the tensile properties of the prepared new materials were tested first.

The basic tensile characteristics are shown in Table 1.

**Figure 6:**
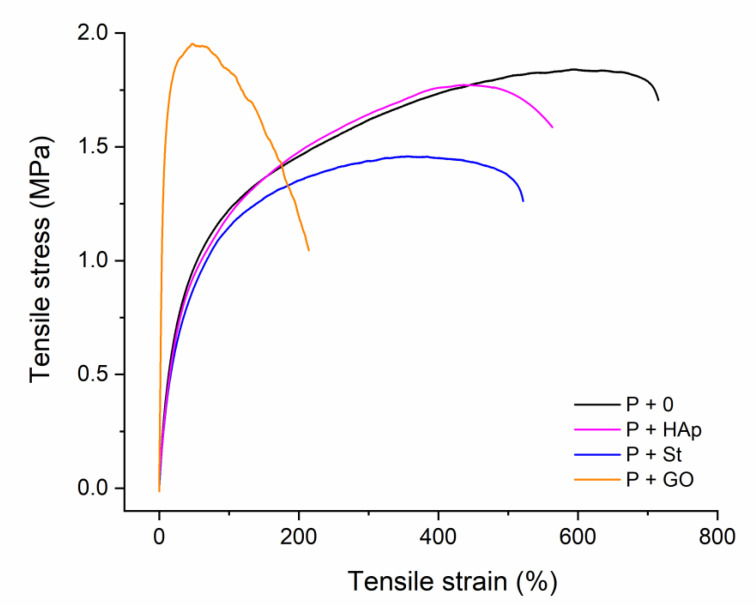
Stress-strain curves of the PUU film and nanocomposite films with starch, graphene oxide and hydroxyapatite.

Stress-strain curves of the selected films, that is, the neat film and the nanocomposite films with starch, graphene oxide and hydroxyapatite, are shown in Figure 6.

Tensile data of the nanocomposites were evaluated and compared with the data of the neat PUU matrix.

Young’s modulus—Almost all nanocomposites have lower values of the Young’s modulus compared to the value of the neat PUU film. This indicates that the total interfacial interactions matrix-filler do not fully compensate hydrogen bonding and dipole-dipole interactions built in the neat PUU film. The film with the graphene oxide is the only exception—Young’s modulus is 5,5 times higher than in the neat matrix. If the shape of the filler is compared, P+CNC, P+MNa, P+HALL films—that is films with higher aspect ratio (needles, platelets, nanotubes)—have the tendency to have a higher Young’s modulus than spherical nanoparticles (P+HAp, P+SiO_2_, P+St). The influence of the organic/inorganic nature on the Young’s modulus was not confirmed.

Stress values—two stress values on the stress-strain curve are detectable—Stress at the yield-point (tensile strength) and stress-at-break. Film with graphene oxide, P+GO had the highest tensile strength of all films prepared. Nanocomposite with hydroxyapatite, P+HAp, has identical tensile strengths to neat PUU matrix; the tensile strength gradually decreases for film with halloysite, silica, Cloisite Na, starch and nanocellulose. Tensile strength values of organically-filled nanocomposites are of slightly lower values than of inorganically-filled analogues.

Values of stress-at-break differ. Film with the graphene oxide has the lowest stress-at-break probably due to relatively continuous arrangement in the nanocomposite (see Section 3.3, TEM analysis). In this case, the overall surface between the matrix and filler is substantially higher than in other nanocomposites. Inorganically filled film have the tendency of higher stress-at-break as compared to organically filled analogues. As fillers in all nanocomposites are present in the form of either isolated particles or their aggregates (see Section 3.4, TEM analysis) the influence of original size and shape is or need not be so pronounced, because the overall interfacial interactions PUU matrix-filler are or appear similar.

Elongation values—two elongation values were measured—elongation at the yield point (i.e., the elongation value at the tensile strength) and elongation-at-break. The highest elongation at the yield point was found for the neat PUU film and the lowest for the film filled with the graphene oxide. Organically-modified nanocomposites have slightly lower values of elongation at the yield point than all inorganic nanocomposites. Smaller spherical filler particles (P+SiO_2_ and P+St) films have lower values than particles with higher size of particles (agglomerates) in the nanocomposite, not depending on the shape (Cloisite, halloysite, hydroxyapatite). Elongation-at-break has the identical trend as elongation in the yield point.

Toughness is expressed as the energy-to break the sample per volume unit. It relates to the area under the stress-strain curve. Toughness values of nanocomposite film increase in the order—P+GO, P+CNC, P+SiO_2_, P+St, P+MNa, P+HALL, P+HAp (P+0). It follows from this dependence that the total area of the interface filler-matrix is dominant. As the values decrease with the area (i.e., possible intensity of interface interactions) it can be concluded that total PUU-filler interactions in all nanocomposites do not compensate physical bonds in the neat PUU matrix. In this way, the interface interaction matrix-filler can be considered as relatively weak.

Tensile characteristics of the nanocomposites were found slightly deteriorated as compared to the neat PUU matrix, due to different total PUU-PUU and PUU-filler interactions. Nevertheless, the relatively weak interfacial interaction matrix-filler can be beneficial for the preparation of degradable materials (see Section 3.6).

### 3.4. TEM Analysis

**Figure 7:**
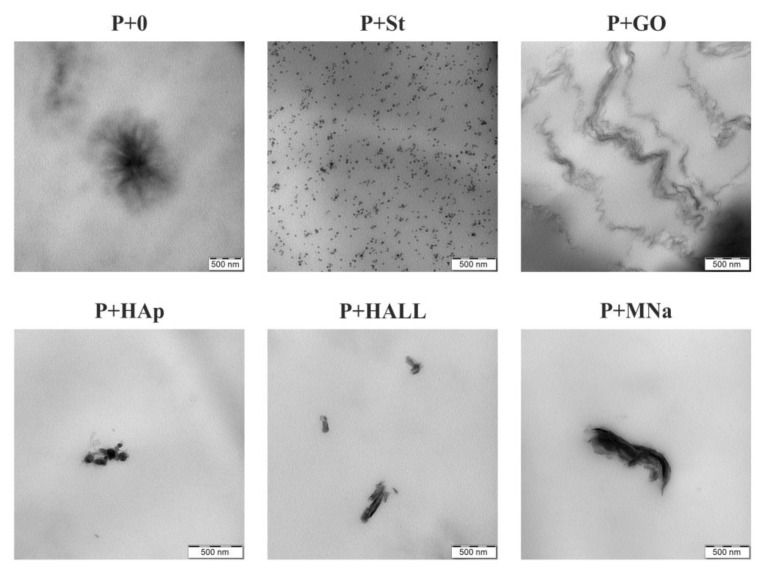
Transmission electron microscopy (TEM) images of the neat PUU matrix (P+0) and selected PUU nanocomposites.

The inner arrangement of the neat PUU film and the filler assembly in the PUU nanocomposites were studied by TEM analysis. The results are shown in Figure 7. The neat PUU matrix was mostly amorphous but spherical semi-crystalline formations (spherulites of size approximately 1 µm in diameter) were detected as well. This result shows the strong tendency of submicron-PUU particles to self-assemble into organized micrometre-sized structures. This phenomenon was recently observed in similar PU and PUU films [36].

The filler assembly in the PUU matrix is likely responsible for the functional properties of nanocomposite films. Comparing their tensile properties, the GO-filled sample substantially differed from the other samples (see Section 3.3). This difference is caused by the ‘fibre-like’ GO arrangement in the PUU matrix forming a continuous connection, unlike the other nanocomposites, in which individual particles and particle aggregates are dispersed and separated throughout the PUU matrix (Figure 7). Other details of the GO morphology are shown in the Supplementary results (text and Figure S3).

The starch behaviour was very interesting. The starch nanoparticles in the nanocomposite were small, relatively uniform (average ca. 20 nm) and homogeneously dispersed within the matrix (Figure 7). This observation can be explained by taking into account the preparation procedure of the starch-containing nanocomposite—heating at 65 °C for 20 min under rigorous stirring. Original starch grains are micron-sized units (Figure S3) and are probably broken up into smaller particles in the heating process (´solubility´ process). PUU particles prevented the reagglomeration of starch during the P+St film preparation, resulting in a transparent material (Figure 2). A favourable starch arrangement is generally very important for the preparation of (bio)degradable nanocomposite coatings from waterborne PU (PUU) dispersions.

Despite their different original shapes and sizes, hydroxyapatite, halloysite, montmorillonite, silica and nanocellulose particles showed a similar behaviour in their TEM images. All of them formed irregular assemblies of nm to µm size surrounded by the PUU matrix (Figure 7; P+SiO_2_ and P+CNC are not shown). The spherulites in the nanocomposites were either not detected or were strongly defective. This result suggests that the nanofillers acted as a barrier for the self-assembly of the PUU particles into organized structures in the resulting films.

### 3.5. FTIR Spectroscopy

**Figure 8:**
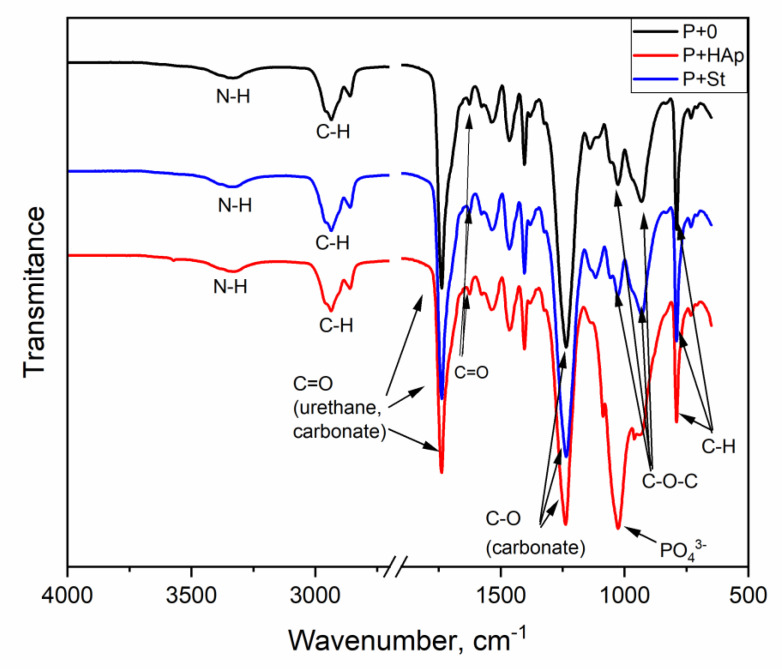
Fourier transform infrared (FTIR) spectra of selected films: neat matrix (P+0), nanocomposite with starch (P+St) and hydroxyapatite (PU+HAp).

The FTIR spectra confirmed the anticipated structures in the neat PUU and PUU nanocomposite films. The FTIR spectra did not differ significantly from each other, with the exception of hydroxyapatite having a significant peak (PO_4_)^3−^ at 1026 cm^−1^ (Figure 8). The characteristic peaks and their assignments are shown in Table 2. FTIR analysis of the selected films is further discussed in Section 3.7.

### 3.6. Thermogravimetric Analysis

Thermogravimetric analysis was used to test the thermal stabilities of the neat PUU film and the nanocomposite films. No significant differences were observed in the thermal degradation of the different samples. All the films were thermally stable up to at least 200 °C, with *T*_5%_ varying in range (270 ± 6) °C without any systematic trend (Table 3, 2nd column).

All the PU films had two derivative thermogravimetry (DTG) maxima, except the sample with graphene oxide, which only had one DTG maximum (Table 3, 3rd and 4th columns). The neat poly(urethane-urea) degraded in two stages—the first one occurred in the temperature range of 207 to 375 °C (with *DTG*_max1_ at 303 °C) and the second one occurred between 401 and 447 °C (with *DTG*_max2_ at 420 °C). The PU films with graphene oxide and hydroxyapatite (*DTG*_max1_ at 336 °C and 335 °C, respectively) had higher thermal stability than neat PUU. The PU film with montmorillonite degraded at a slightly lower temperature than the neat PUU (*DTG*_max1_ at 290 °C) but the second degradation step was shifted to a higher temperature (*DTG*_max2_ at 443 °C).

The char content at 500 °C was low, from 0.6 wt% in the neat PUU up to 2.3 wt% in the starch-filled film (Table 3, 5th column). No dependence of the char content on the nanofiller nature and shape was found.

### 3.7. Study of the Ability of the Film to Degrade

The neat PUU film and all the nanocomposite films were tested in terms of their degradation ability. Accelerated model in vivo degradation tests were used, that is, the samples were incubated in a H_2_O_2_ + CoCl_2_ solution at 37 °C for up to 21 days.

The results are shown in Table 4. The degradation is given as the mass loss caused by H_2_O_2_ + CoCl_2_ exposure. The lowest mass loss was detected for the neat PUU film after incubation of up to one week. After three weeks, the mass loss was comparable with the halloysite and starch nanocomposites. The degradation of the nanocomposite films can be roughly divided into three groups with an increasing amount of degradation—spherical nanofillers (silica, starch, hydroxyapatite), platelets/nanotubes (montmorillonite, halloysite) and thin sheets/needles (graphene oxide, nanocellulose). The nanofiller shape was thus found to be the key factor in the degradation process. All the films were sticky after degradation, especially after 21 days.

Starch- and hydroxyapatite-containing nanocomposites after 21 days of degradation were subjected to further FTIR and TGA analyses.

**Figure 9:**
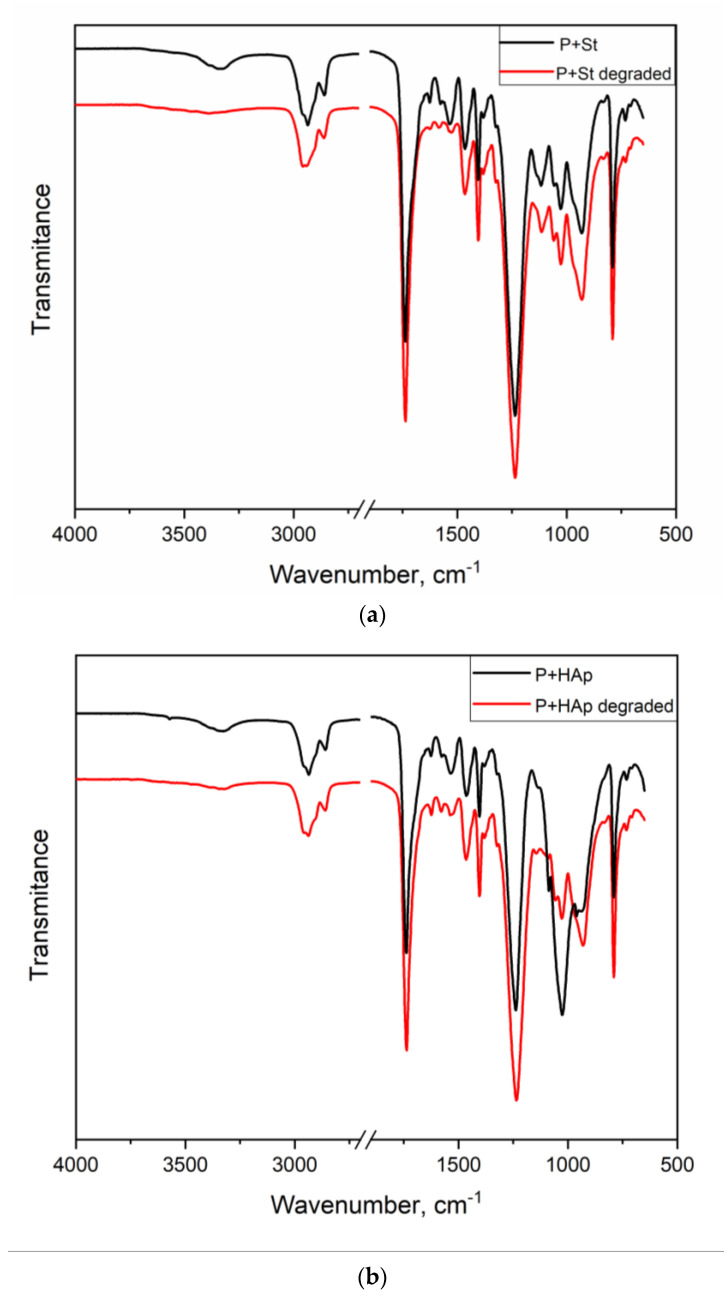
Comparison of the FTIR spectra of the P+St (**a**) and P+HAp (**b**) films before (red) and after 21 days of degradation (black).

Figure 9 shows the FTIR spectra of the untreated and degraded films. Although the spectra were very similar, several regions indicated structural changes.

Although the spectra were complex due to the complex PUU structures, distinct differences were observed in the P+St spectrum at approximately 1540, 1620 and 3303 cm^−1^ and at approximately 930, 1026, 1540 and 3320 cm^−1^ in the spectrum of the P+HAp film. These differences indicated that the degradation process affected C-O-C (carbonate), NH (urethane, urea), C=O (urea, strongly bonded) and H-bonded NH (urethane, urea, event biuret, amide) structures; cf. Table 2. In other words, the structures contributing to strong hydrogen bonds were partially destroyed during the degradation process. The substantial decrease in the peak at 1026 cm^−1^ in P+HAp proves HAp leaching from the nanocomposite signalizing relatively weak interfacial interactions PUU matrix-HAp particles in P-HAp nanocomposite.

**Figure 10:**
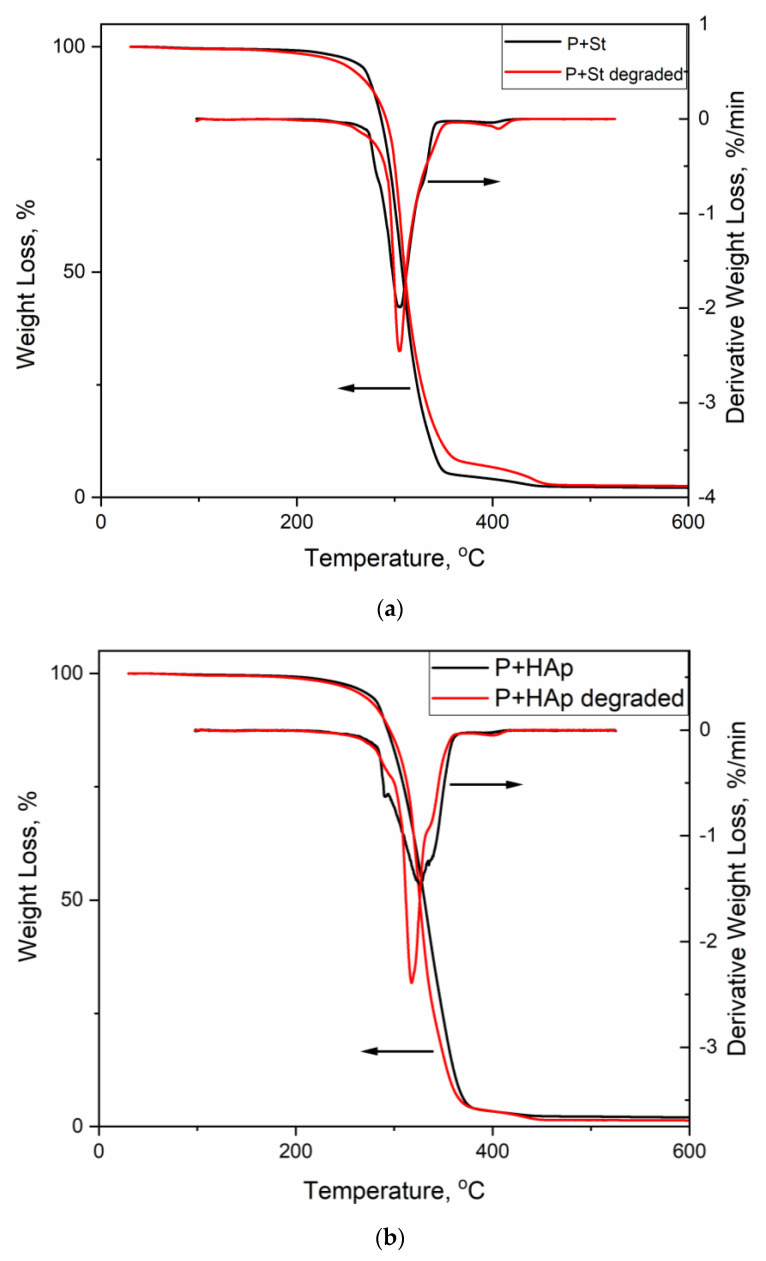
Comparison of the thermogravimetric analysis (TGA) curves of the P+St (**a**) and P+HAp (**b**) films before (red) and after 21 days of degradation (black). Weight loss—left scale, derivative weight loss—right scale.

Figure 10 shows the representative TGA dependences of the P+St and P+HAp films before and after degradation. No substantial changes in their thermal curves were found. Although the degradation process was apparent (Table 4, Figure 9), it did not significantly influence the thermal stability of the material. Both degraded materials are thermally stable up to 200 °C in the minimum, similar to analogues not degraded.

Further details of the degradation analyses are given in the Supplementary results.

## 4. Conclusions

^1^H NMR spectroscopy confirmed the proposed reaction scheme of the poly(urethane-urea) nanoparticle preparation using an innovative technique. NMR analysis also clarified why some procedures led to the successful preparation of colloidal particles, while other procedures resulted in gelation.

Analysis of the waterborne PUU matrix and the PUU nanocomposites confirmed that a broad choice of nanofillers can be used for the preparation of mostly-transparent waterborne PUU-based films.

The internal arrangement of the nanofiller in the PUU matrix was the dominant factor affecting the functional properties of waterborne nanocomposite films. The original nature of the nanofiller (organic/inorganic) seemed to be not significant. All the nanofillers slightly reduced the tensile properties because they are barrier for the PUU nanoparticle re-arrangement into the film during slow water evaporation. However, most of the nanofillers slightly increased the thermal stability of the nanocomposites. The nanocomposites showed different responses to the degradation process. The lowest mass loss caused by degradation was found for small spherical particles, that is fillers with low aspect ratio (starch, hydroxyapatite), while thin sheets or needles, that is, fillers with high aspect ratio (nanocellulose, graphene oxide) led to total film break-up. This phenomenon is very important from a practical perspective, because the degree of the degradation susceptibility of waterborne nanocomposites can be controlled by the shape (aspect ratio) of the nanofiller.

From the set of nanocomposites prepared, the most useful ones seemed to be the transparent nanocomposite with starch (as biobased, relatively stable material) or with nanocellulose (as degradable film). The film with graphene oxide is the representative of non-transparent degradable material. All the nanocomposites are promising materials usable as top coating materials in human-body conditions.

As waterborne PUU and all the PUU-nanofiller dispersions contain submicron and micron-sized particles, the preparation of thin films by the spraying procedure may be beneficial. Preliminary tests of transparent compact thin film preparation by spraying technique on glass and polymer substrates are very promising.

## Figures and Tables

**Table 1:** Tensile characteristics of the neat poly(urethane-urea), PUU and PUU nanocomposite films.

Nanofiller	Young’s Modulus, *E* (MPa)	Stress-at-Break, *σ*_b_ (MPa)	Elongation-at-Break, *ε*_b_ (%)	Energy-at-Break, Toughness (mJ·mm^−3^)
P+0	5.3 ± 0.3	1.6(1.8 *) ± 0.1(0.1 *)	722(601 *) ± 31(20 *)	11.4 ± 1.0
P+SiO_2_	3.9 ± 0.3	1.3(1.6*) ± 0.1(0.1 *)	510(393 *) ± 16(16 *)	7.1 ± 0.5
P+ HAp	4.0 ± 0.2	1.5(1.8 *) ± 0.1(0.1 *)	583(463 *) ± 62(51 *)	9.1 ± 1.3
P+MNa	4.4 ± 0.3	1.4(1.6 *) ± 0.1(0.1 *)	547(415 *) ± 32(28 *)	8.0 ± 0.5
P+HALL	4.1 ± 0.2	1.1(1.7 *) ± 0.1(0.1 *)	559(446 *) ± 47(58 *)	8.8 ± 1.4
P+St	3.8 ± 0.1	1.2(1.5 *) ± 0.1(0.1 *)	515(373 *) ± 48(25 *)	6.9 ± 0.7
P+CNC	4.4 ± 0.5	1.0(1.4 *) ± 0.1(0.1 *)	471(310 *) ± 39(12 *)	5.8 ± 0.7
P+GO	31.0 ± 1.4	0.9(1.9 *) ± 0.1(0.1 *)	49(205 *) ± 4(11 *)	3.4 ± 0.2

* Values of stress (strain) at the yield point. The yield was set as the first point on the stress-strain curve at which the slope decreases to zero.

**Table 2:** Characteristic peaks, bonds and vibration modes in PUU films.

Wavenumbers (cm^−1^)	Bonds	Vibration Modes
3360–3335	N-H (urethane, urea, event. biuret, amide) H bonded	Stretching
2900–2850	C-H (in CH_2_ and CH_3_)	Asymmetrical and symmetrical stretching
1741–1738	C=O in urethane free	Stretching
1626–1623	C=O in urea strongly bonded (ordered/bidentate)	Stretching
ca 1535	N-H in urethane and urea	Symmetrical bending
ca 1464	C-N in urethane and urea	Symmetrical stretching
ca 1460	CH in CH_2_	Symmetrical bending
ca 1240	O-C=O in carbonate	Antisymmetrical stretching
ca 1240	N-H in urethane and urea	Asymmetrical bending
ca 1240	C-N in urethane and urea	Asymmetrical stretching
ca 1035	C-O-C in urethane	Stretching
ca 1010	(PO_4_)^3-^ in hydroxyapatite	Symmetrical stretching
ca 940	C-O-C in carbonate	Symmetrical stretching

**Table 3:** Thermal properties of the PUU matrix and the nanocomposite films.

Nanofiller	*T*_5%_ (^o^C)	*T*_DTGmax1_ (^o^C)	*T*_DTGmax2_ (^o^C)	Char at 500 ^o^C (wt%)
P+0	272	303	420	0.6
P+SiO_2_	267	311	422	1.8
P+HAp	276	335	422	2.2
P+MNa	264	290	443	1.5
P+HALL	272	312	421	2
P+St	269	306	429	2.3
P+CNC	270	306	433	1.4
P+GO	272	336	-	1.8

**Table 4:** Mass loss values after accelerated in vivo degradation process.

Film	Mass Loss After 2 Days, wt%	Mass Loss After 7 Days, wt%	Mass Loss After 21 Days, wt%
P+0	1.8	2.2	12.8
P+SiO_2_	2.9	3.1	7.4
P+HAp	3.0	4.6	14.9
P+MNa	4.0	5.4	19.5
P+HALL	3.8	7.1	11.9
P+St	3.1	3.6	10.6
P+CNC	5.7	33.9	Broke apart
P+GO	10.9	58.9	Broke apart

## Supplementary Materials

The following are available online at https://www.mdpi.com/2073-4360/12/9/2001/s1, Figure S1: 1H NMR spectrum of G4672 macrodiol measured in deuterated acetone at 22 °C. *and** are the methylene protons of the macrodiol end-groups terminated by hydroxyls., Figure S2: 1H NMR spectrum of the reaction mixture before starting the polyaddition reaction measured at 22 °C using deuterated DMF as the external solvent (in a capillary), Figure S3: TEM image of starch grains (left) and AFM image of graphene oxide sheet (right).Table S1: Details of the nanofillers used.
