# Supplementary material for: The Influence of Nanofiller Shape and Nature on the Functional Properties of Waterborne Poly(urethane-urea) Nanocomposite Films
Source: Polymers (Basel). 2020 Sep 2;12(9):2001. doi: 10.3390/polym12092001 (PMC7565782; doi:10.3390/polym12092001)
Supplement: Supplementary file 1 [file polymers-12-02001-s001.pdf]

The influence of nanofiller shape and nature on the functional properties of waterborne poly(urethane-urea) nanocomposite films

Supplementary results

Milena Špírková, Jiří Hodan, Rafał Konefał, Luďka Machová, Pavel Němeček and Aleksandra Paruzel

Institute of Macromolecular Chemistry CAS, Heyrovského nám. 2, 162 06 Prague 6, *Czech Republic* (*www.imc.cas.cz*), * *spirkova@imc.cas.cz*, *+420 296 809 384*

Ad. 2.1. Materials

Table S1. Details of the nanofillers used

Filler (abbreviation)	Nature*	State**	Shape/ AR***	Simplified Formula	Producer	Total time treatment
Ludox AS-40 (**SiO_2**)	I	D 40 wt%	spherical ca 35nm/1	SiO_2	Aldrich	1 day
Hydroxyapatite **(HAp)**	I	D 10 wt%	spherical ≤ 200 nm/1	$Ca_5(PO_4)_3$ (OH)	Sigma Aldrich	3 days
Cloisite® Na (**MNa**)	I	P	platelets /up to 10	2:1 phyllosilicate	Southern Clay Products, Inc.	2 days
Halloysite (**HALL**)	I	P	Nanotubes/ up to10	$Al_2Si_2O_5 (OH)_4 \cdot 2H_20$	Sigma-Aldrich	3 days
Starch soluble (**St**)	O	P	grains 5-10 µm/ 1	$(C_6H_{10}O_5)_n$	Sigma-Aldrich	1 day, 20 min at 65 ºC
Nanocellulose (**CNC**)	O	D 8 wt%	needle/ 20-30	$(C_6H_{10}O_5)_n$	Blue Goose Biorefineries Inc.	7 days
Graphene oxide (**GO**)	O	D 1.3 wt%	platelets/ up to 350	C (+O+H)	Synthesized Hummers method [33]	5 days

* I= inorganic, * O = organic; ** D = dispersion; ** P = powder; *** AR = estimated aspect ratio

Estimated aspect ratio is based on the producer info and our previous and current experiments.

Ad 3.1. NMR spectroscopy

Ad (i): ^{1}H NMR spectra of PCD measured in deuterated acetone with signal assignments are shown in Figure S1. The spectrum shows the characteristic chemical shifts of the protons in the –O**CH**$_2$-hexane unit (**1, 6**) and the butane unit (**7, 10**) at δ≈ 4.10 ppm and δ≈ 4.15 ppm, respectively. The signals at δ≈ 1.65 ppm and δ≈ 1.75 ppm are attributed to the –OCH$_2$**CH**$_2$- protons of the hexane- (**2, 5**) and butane-repeating units (**8, 9**). Moreover, the spectrum shows a signal of the –O–CH$_2$CH$_2$**CH**$_2$- (**3, 4**) group in the hexane unit at δ≈ 1.40 ppm. The signals corresponding to the methylene protons of the end-groups HO-**CH**$_2$- (*) and HO-CH$_2$**CH**$_2$- (**) from both monomer-repeating units are assigned to the peaks at δ≈ 3.55 ppm and δ≈ 1.55 ppm, respectively. The butane/hexane molar ratio of the copolymer, estimated from the relative intensity of the respective signals (7,10):(1,6), is 5:3, which corresponds to a ratio of 62.5 to 37.5 mol % of hexane-to-butane building units. This ratio is consistent with the 7:3 molar ratio of hexane-to-butane units specified by the producer.

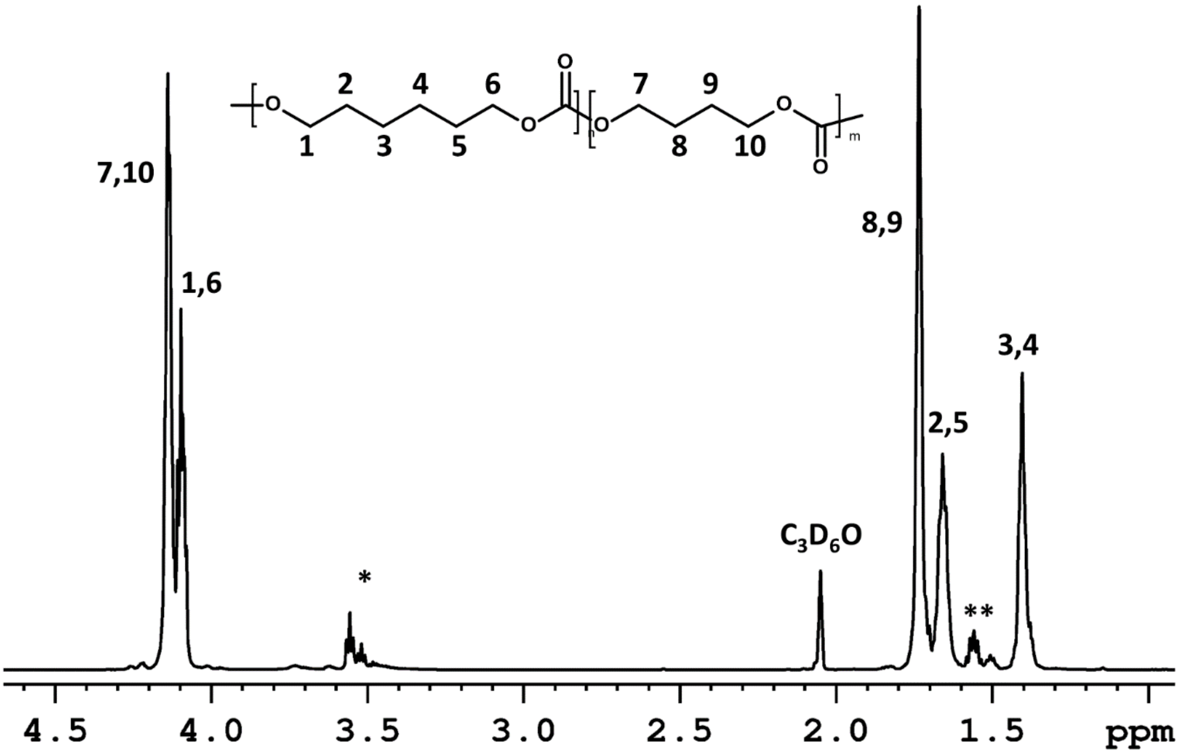

Figure S1. ^{1}H NMR spectrum of G4672 macrodiol measured in deuterated acetone at 22 °C. *and** are the methylene protons of the macrodiol end-groups terminated by hydroxyls.

Ad (ii): In the next step of investigation, the reaction mixture before starting the polyaddition reaction composed of PCD, hexamethylene diisocyanate (HDI), 2,2-bis(hydroxymethyl)propionic acid (DMPA), DBTDL and acetone was measured by ^{1}H NMR spectroscopy using deuterated DMF-$_{d7}$ as the external solvent (in a capillary). The resulting spectrum is shown in Fig. S2. In addition to the signals of PCD described above, peaks attributed to DMPA methylene (**14**) and methyl (**15**) groups are observed at δ≈ 3.70 ppm and δ≈ 1.15 ppm, respectively. Furthermore, the remaining signal in the spectrum at δ≈ 3.38 ppm can be assigned to the OCN-**CH**$_2$- methylene group (**11**) of HDI. The peaks of the other two methylene groups OCN-CH$_2$-**CH**$_2$- (**12**) and OCN-CH$_2$-CH$_2$-**CH**$_2$- (**13**) overlap with the (**2,5**) and (**3,4**) signals of PCD.

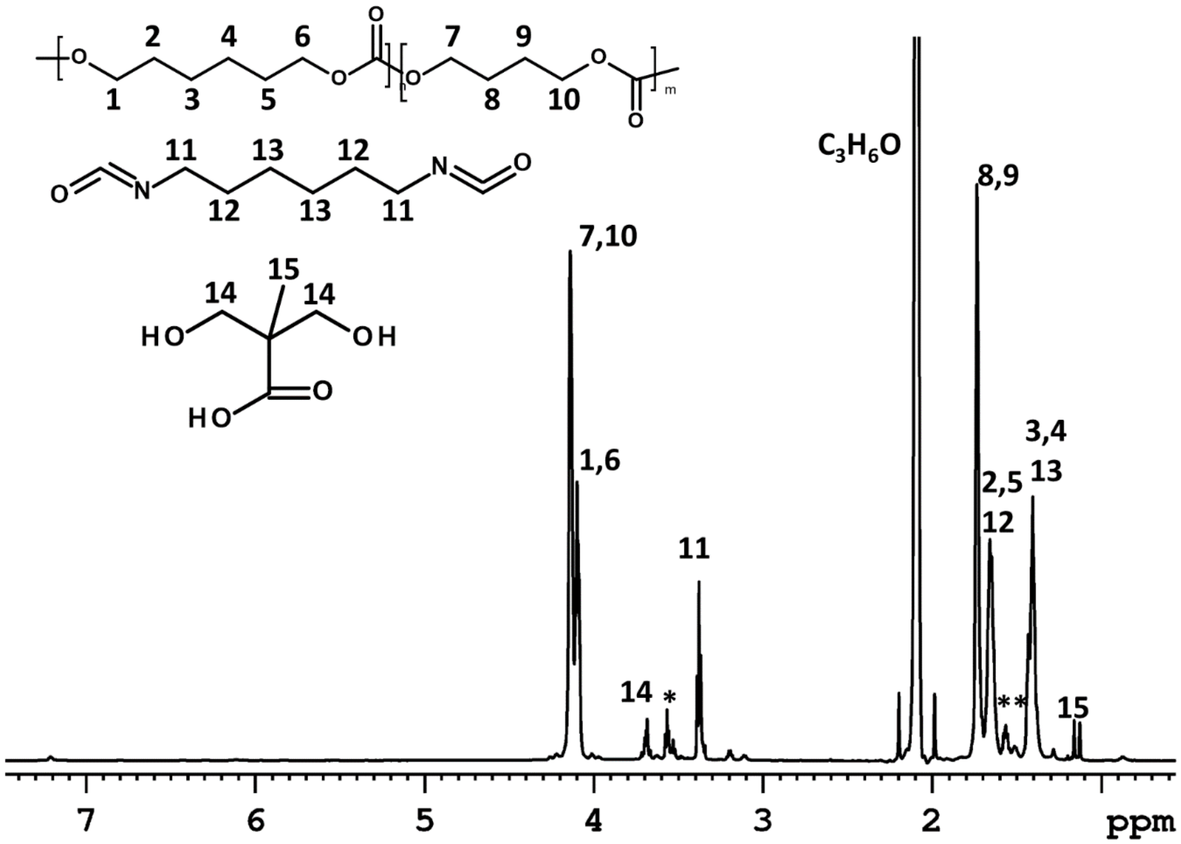

Figure S2. ^{1}H NMR spectrum of the reaction mixture before starting the polyaddition reaction measured at 22 °C using deuterated DMF as the external solvent (in a capillary).

Ad 3.3. TEM analysis

To confirm the starch arrangement in the nanocomposite, neat starch grains were analysed by TEM and compared with the TEM images of the P+St film (Figure 8). A TEM image of the neat starch is shown in Figure S3, left. Spherical particles with diameters of 5 to 10 μm were detected.

AFM analysis of GO was used as a supplementary analysis tool to TEM. It was found that the sizes of the individual GO particles ranged from hundreds of nm up to units of microns in length and width. Figure S3 (right) shows an example of a GO particle: the length is ca. 550 nm, the width is ca. 300 nm, and the thickness is ca. 1.5 nm. The strong 2D-character of the GO sheets is probably responsible for the different behaviour of the P+GO film compared to the other prepared materials.

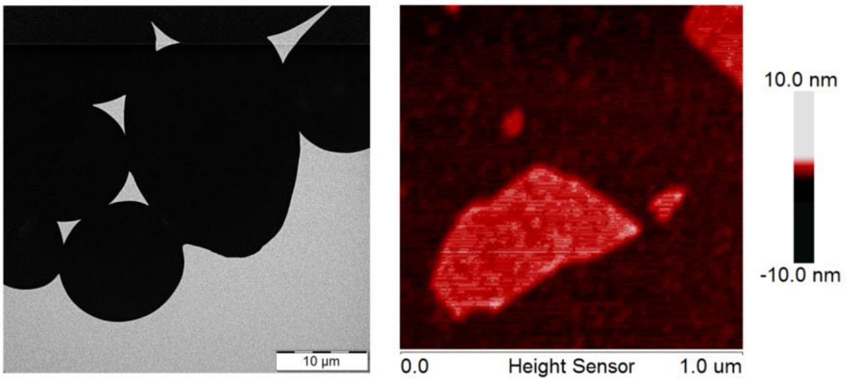

Figure S3. TEM image of starch grains (left) and AFM image of graphene oxide sheet (right).

Ad 3.6. Study of the ability of the film to degrade

Degradation experiments confirmed that the nanofiller shape influences the efficiency of the degradation. It is probably the consequence of the nanocomposite film formation: The film is constituted from two kinds of nanoparticles; PUU and filler components. The compact film is gradually formed by slow water evaporation via different physical PUU-PUU, PUU-filler and filler-filler particle interactions, van der Waals forces included. As tensile characteristics of the neat PUU matrix was the best of all samples, the physical PUU-PUU interactions appear to be the strongest of all kind of interactions. Total PUU-filler interfacial interactions should be weaker.

The overall area of PUU-filler interactions is the factor influencing the nanocomposite compactness. And it significantly differs for different shapes (aspect ratios). One example based on TEM images: if two model particles are compared: Sheet GO of size 550 nm x 300 nm x 1.5 nm has the volume 2.47 x 10^5 nm^3 and total surface area 3.32 x 10^5 nm^2. Model starch spherical nanoparticle of diameter 20

nm has the volume 4.19 x 10^3 nm^3 and total surface area 1.26 x 10^3 nm^2. While the volumes of both model particles differ 59 times, the total surface areas differ 263 times. These differences, together with size and shape of pertinent agglomerates could explain the different susceptibility of both nanocomposites to the degradation.

PUU matrix has the tendency for formation of highly organized structures of micrometre size (spherulites, as shown in Fig. 7) within mostly amorphous PUU state. The fillers are present in the nanocomposite as individual particles but mostly as agglomerates surrounded by amorphous (continuous) matrix. If the particles originally differentiated in the shape and size are organized in the nanocomposite to the formations of similar shape and comparable size (e.g., P+HALL, P+MNa and P+HAp; see Figure 7), their degradation behaviour is similar, see Table 4.

Nanocomposites with small spherical particles are the most resistant materials to the degradation process.
